# Reference gene identification for reliable normalisation of quantitative RT-PCR data in *Setaria viridis*

**DOI:** 10.1186/s13007-018-0293-8

**Published:** 2018-03-21

**Authors:** Duc Quan Nguyen, Andrew L. Eamens, Christopher P. L. Grof

**Affiliations:** Centre for Plant Science, School of Environmental and Life Sciences, University of Newcastle, University Drive, Callaghan, NSW 2308 Australia

**Keywords:** *Setaria viridis*, C_4_ crop, Quantitative RT-PCR normalisation, Reference genes, Gene expression

## Abstract

**Background:**

Quantitative real-time polymerase chain reaction (RT-qPCR) is the key platform for the quantitative analysis of gene expression in a wide range of experimental systems and conditions. However, the accuracy and reproducibility of gene expression quantification via RT-qPCR is entirely dependent on the identification of reliable reference genes for data normalisation. Green foxtail (*Setaria viridis*) has recently been proposed as a potential experimental model for the study of C_4_ photosynthesis and is closely related to many economically important crop species of the Panicoideae subfamily of grasses, including *Zea mays* (maize), *Sorghum bicolor* (sorghum) and *Sacchurum officinarum* (sugarcane). *Setaria viridis* (Accession 10) possesses a number of key traits as an experimental model, namely; (i) a small sized, sequenced and well annotated genome; (ii) short stature and generation time; (iii) prolific seed production, and; (iv) is amendable to *Agrobacterium tumefaciens*-mediated transformation. There is currently however, a lack of reference gene expression information for *Setaria viridis* (*S. viridis*). We therefore aimed to identify a cohort of suitable *S. viridis* reference genes for accurate and reliable normalisation of *S. viridis* RT-qPCR expression data.

**Results:**

Eleven putative candidate reference genes were identified and examined across thirteen different *S. viridis* tissues. Of these, the geNorm and NormFinder analysis software identified *SERINE*/*THERONINE*-*PROTEIN PHOSPHATASE 2A* (*PP2A*), *5*′-*ADENYLYLSULFATE REDUCTASE 6* (*ASPR6*) and *DUAL SPECIFICITY PHOSPHATASE* (*DUSP*) as the most suitable combination of reference genes for the accurate and reliable normalisation of *S. viridis* RT-qPCR expression data. To demonstrate the suitability of the three selected reference genes, *PP2A*, *ASPR6* and *DUSP*, were used to normalise the expression of *CINNAMYL ALCOHOL DEHYDROGENASE* (*CAD*) genes across the same tissues.

**Conclusions:**

This approach readily demonstrated the suitably of the three selected reference genes for the accurate and reliable normalisation of *S. viridis* RT-qPCR expression data. Further, the work reported here forms a highly useful platform for future gene expression quantification in *S. viridis* and can also be potentially directly translatable to other closely related and agronomically important C_4_ crop species.

**Electronic supplementary material:**

The online version of this article (10.1186/s13007-018-0293-8) contains supplementary material, which is available to authorized users.

## Background

C_4_ plants include some of the most productive species in the world due to their unique leaf anatomy that supports efficient fixation of CO_2_ into biomass and their adaptation to high temperature and intense light [1]. Recently, *Setaria viridis* (*S. viridis*), a C_4_ monocot grass, has emerged as a promising experimental model to study the photosynthesis and cell wall biology pathways unique to C_4_ plants. *Setaria viridis* (green foxtail) is the wild ancestor of the minor crop foxtail millet (*S. italica*) and has close evolutionary links to several major C_4_ crop species, including *Zea mays* (maize), *Sorghum bicolor* (sorghum) and *Saccharum officinarum* (sugarcane), as well as the bioenergy grasses *Panicum virgatum* (switchgrass) and Miscanthus × giganteus (the perennial hybrid of *Miscanthus sinensis* and *Miscanthus sacchariflorus*), all of which belong to the Panicoideae subfamily of Poaceae grasses [2]. *Setaria viridis* has been identified as a model species as it possesses many desirable traits, including; (i) a small sized (~ 500 Mb), sequenced and well annotated genome; (ii) short stature (20–30 cm in height) and rapid life cycle (6–9 weeks); (iii) prolific seed production (13,000 seeds/plant), and; (iv) is amendable to *Agrobacterium tumefaciens*-mediated transformation [3]. Use of *S. viridis* as an experimental model is further supported by the rapidly expanding availability of genetic tools and online genomic information [4–6].

Recent advances in the quantitative real-time polymerase chain reaction (RT-qPCR) approach have revolutionised gene expression studies. The RT-qPCR approach provides greatly enhanced levels of sensitivity and accuracy for gene expression analysis compared to the more conventional methods, semi-quantitative RT-PCR and northern blot hybridisation [7]. The RT-qPCR approach is reliant upon the exponential incorporation of fluorescent dyes into amplified products [8], and has become the method of choice to validate RNA sequencing (RNA-Seq) data, or to quantify the abundance of a select group of target genes stemming from a large population of expressed transcripts [9, 10]. The accuracy and reliability of gene expression data derived from RT-qPCR experiments is not only influenced by the quality and quantity of the RNA used as template for complementary DNA (cDNA) synthesis, but is highly dependent on normalisation to one or more stably expressed reference genes. To date, the lack of a stably expressed reference gene(s) for the accurate normalisation of *S. viridis* RT-qPCR data has been a major hurdle for gene expression studies in this species.

The selection of a subset of reference genes for normalisation of target gene expression is based on the identification of gene transcripts that are constantly expressed at a high level throughout plant development, or in a specific tissue or organ under analysis [11, 12]. Traditionally, housekeeper genes are preferred for RT-qPCR data normalisation as the protein products encoded by these genes are required for maintenance of fundamental cellular metabolism. Further, due to their assigned function, the expression of a housekeeper gene is assumed constant irrespective of the organ or tissue type under analysis, or the developmental stage or physiological condition(s) of the assessed species [13, 14]. Several housekeeper genes have been widely used in *Arabidopsis thaliana* (*CYCLOPHILIN* (*CYC*), *ACTIN2* (*ACT2*) and *ELONGATION FACTOR 1*α (*EF1*α; [15, 16])), rice (*ACTIN1* (*ACT1*) and *UBIQUITIN5* (*UBI5*; [7, 11])), and sorghum (*SERINE*/*THERONINE*-*PROTEIN PHOSPHATASE 2A* (*PP2A*) and *EUKARYOTIC INITIATION FACTOR 4α* (*EIF4α*; [14])). However, a growing body of research has shown that housekeeper genes cannot be utilised universally across different plant species, experimental conditions, or even across different developmental stages within a single species [9, 11, 14]. Further, normalisation of RT-qPCR data using just a single housekeeper gene is no longer recommended [17, 18], as such an approach could result in a biased or incorrect interpretation of the expression of the studied gene(s). Therefore, identification of a group of reference genes, which are constitutively and stably expressed across different tissues, organs, developmental stages and experiment conditions, is the first critical step for the completion of accurate and unbiased (reliable) quantification of gene expression by RT-qPCR [19]. For this reason, many statistical tools (for example; NormFinder, geNorm, BestKeeper), which are publically available online, have been developed to identify cohorts of suitable reference genes for RT-qPCR data normalisation.

Here, in order to advance the current quantification of gene expression in *S. viridis*, we assessed the performance of eleven candidate reference genes across thirteen different tissues to identify a suitable cohort of stably expressed reference genes for the accurate and reliable normalisation of *S. viridis* RT-qPCR expression data. The eleven normalisation candidates assessed were identified from two RNA-Seq datasets, namely an elongating internode dataset [4] and an expanding leaf dataset [5], in combination with those reported previously for RT-qPCR analyses in other C_4_ species [9, 14]. NormFinder and geNorm analyses of candidate reference gene performance identified three candidates, *SERINE*/*THERONINE*-*PROTEIN PHOSPHATASE 2A* (*PP2A*), *5*′-*ADENYLYLSULFATE REDUCTASE 6* (*ASPR6*) and *DUAL SPECIFICITY PHOSPHATASE* (*DUSP*), as the most suitable combination of reference genes for the accurate and reliable normalisation of target gene expression across *S. viridis* tissues. We next used the identified reference gene combination to normalise the expression of seven *S. viridis CINNAMYL ALCOHOL DEHYDROGENASE* (*CAD*) genes. Normalisation of *CAD* gene expression with *PP2A*, *ASPR6* and *DUSP* readily demonstrated the suitably of the three selected reference genes for the accurate and reliable normalisation of *S. viridis* RT-qPCR data across architecturally and developmentally distinct tissues. Together, the data reported in this study forms a highly useful platform for the future quantification of gene expression in *S. viridis*.

## Methods

### Plant material

*Setaria viridis* (L.) Beauv. (Accession A10) was used for all reported experimental work. *Setaria viridis* seeds were sown under a thin layer of a soil mixture composed of coarse sand, coco peat, and perlite at a ratio of 2:1:1. Post seed germination, *S. viridis* plants were cultivated under a controlled growth regime of 16 h light (~ 600 μmol m^−2^ s^−1^) and 8 h dark with a day/night temperature of 28 °C/20 °C. Plant material was harvested at different stages of development. Internode and leaf material was harvested at the 50% ear emergence stage, 20–25 days after germination (DAG), immediately frozen in liquid nitrogen, and stored at -80 °C until required for processing. Of the plant material sampled for processing; (i) whole internode 4, and leaf 4, represented fully elongated mature tissues; (ii) whole internode 5, and leaf 5, represented elongating tissues, and; (iii) whole internode 6, and leaf 6, were sampled to represent immature tissues. Additional representatives of the internode 5 were also sampled and subsequently divided into four developmentally distinct zones upon collection, including the; (i) meristematic (MS); (ii) cell expansion (CEZ); (iii) transitional (TZ), and (iv) mature (MatZ) zones. Whole inflorescence stems were also sampled at the 50% ear emergence stage (S1; 20–25 DAG), the flowering stage (S2; 27–32 DAG; once pollen sacs had appeared on spikelets), and the milky dough stage (S3; 40–45 DAG; at the start of grain fill). The three stages harvested represent immature, elongating and fully elongated mature inflorescence tissues, respectively. In total, four biological replicates were collected and each biological replicate contained tissue sampled from five individual plants (see Additional file 1: Figure S1).

### Total RNA extraction and complementary DNA synthesis

Total RNA was extracted using TRIzol^®^ Reagent according to the manufacturer’s instructions (Invitrogen, USA). Any contaminating genomic DNA was subsequently removed using an Ambion™ TURBO™ DNase kit (Life Technologies, USA). Post DNase treatment, total RNA was column purified using a RNeasy Mini Kit (Qiagen, USA). The concentration and purity of extracted total RNA was next assessed with a NanoDrop™ 1000 Spectrophotometer (ThermoFisher Scientific, USA) and only samples with an A260/A280 absorbance ratio in the range of 1.9–2.1 were subsequently used for the synthesis of complementary DNA (cDNA). One microgram (1.0 μg) of DNase-treated and column purified total RNA was used as template for first strand cDNA synthesis with Superscript^®^ III Reverse Transcriptase (ThermoFisher Scientific, USA) and oligo dT_23_ primer. The resulting cDNA was immediately diluted 1:50 in RNase-free dH_2_O and this working stock was stored at − 20 °C until required as template for all PCR-based analyses, including; standard RT-PCR and RT-qPCR analysis.

### Selection of candidate reference genes and the design of RT-qPCR primers

Eleven candidate reference genes were selected for evaluation to determine their suitability for use to normalise gene expression data stemming from *S. viridis* RT-qPCR analyses. Seven candidate reference genes (*ASPR6*, *SEIPIN*, *STK*, *DUSP*, *FBoxD*, *WNK1* and *GRAS*) were selected based on the bioinformatic analysis of two RNA-Seq datasets from *S. viridis* tissues by screening for genes with relatively stable expression (based on reported RPKM and fold change values). The first dataset interrogated, represented four developmentally distinct zones of an elongating internode, internode 5, and included the; MS, CEZ, TZ and MatZ zones [4]. The second dataset analysed, was derived from leaf 3 sampled from a 10 day old plant [5]. The remaining four candidate reference genes selected for detailed assessment in this study, namely *CUL*, *FPGS*, *PGM* and *PP2A*, were included based on their expression profiles reported in previous RT-qPCR studies performed in *S. viridis* [9] and sorghum [14].

The primers for the eleven candidate reference genes were designed using the Primer3 ver4.0 (RRID: nlx_156833) and NCBI Primer-BLAST (RRID: SCR_003095) web-based tools with the following parameters; (1) a GC content of 50–60%; (2) an amplicon size of 60–120 base pairs (bp), and; (3) a melting temperature (Tm) of 60 ± 1 °C. Primer details are listed in Additional file 2: Table S1. For assessment of primer performance (R^2^) and the efficiency of amplification (E), analysis of standard curves was conducted to determine the performance of each pair of primers.

### Quantitative real-time polymerase chain reaction analysis

All RT-qPCRs were carried out on a Rotor-Gene Q machine (Qiagen, USA) using the GoTaq^®^ qPCR Master mix (Promega, USA). Each 10 μL reaction contained; 5.0 μL of 2X GoTaq^®^ qPCR Master mix; 1.0 μL of each primer (10 μM), 1.0 μL of diluted (1:50) cDNA template (~ 15 ng/μL), and 2.0 μL of RNase-free dH_2_O. The cycle program for product amplification was; 1 cycle of 95 °C for 10 min (hot-start activation), followed by 40 cycles of 95 °C for 15 s (denaturation), and 60 °C for 40 s (annealing/extension). The melt curve was generated for each primer pair across the temperature range of 72–95 °C, and with a temperature increment of 1.0 °C each 5-s period. Each primer pair was assessed using four biological replicates and three technical replicates were performed per biological replicate.

### Data analysis and assessment of candidate reference gene performance

The expression stability of each assessed candidate reference gene was evaluated using two different algorithms, namely; (1) NormFinder (RRID: SCR_003387; [20], and; (2) geNorm (RRID: nlx_156922; [21], and with the use of 52 samples (that is; thirteen different tissue types and 4 biological replicates sampled per tissue type). The raw quantification cycle (Cq) values from this RT-qPCR analysis were converted to relative quantities by using the formula, *Q* = *E*^Δ*C*q^, where *E* is the efficiency of amplification, and ΔCq is the difference in quantification cycle of the most lowly expressed target gene and the *C*q value of all samples analysed in the dataset. Candidate reference genes for *S. viridis* RT-qPCR data normalisation were selected based on their NormFinder and geNorm ranked stability values (*M*). The candidate reference gene returning the lowest *M* value was identified as the most stably expressed whereas the reference gene returning the highest *M* value was considered to be the least stably expressed. GeNorm was also used to determine the optimal number of candidate reference genes via pairwise variation (*Vn*/*Vn* + *1*) analysis with the proposed threshold value of 0.15 used as outlined by Vandesompele et al. [21].

## Results and discussion

### Identification of candidate reference genes

Quantitative RT-PCR has become the most accurate, and therefore most frequently used technique for the quantification of transcript abundance in many plant species due to the rapid, high throughput, sensitive and accurate nature of this approach [22]. The success of gene expression quantification via the RT-qPCR approach is however, entirely dependant upon the identification and use of a group of suitable reference genes for expression normalisation due to; (1) there not being any ‘universal’ reference gene(s) available for use across a wide range of phenotypically distinct plant species or experimental conditions in a single species [23, 24], and; (2) the use of only a single reference gene could lead to an incorrect functional interpretation of the expression profile of a gene [18, 21]. Fang et al. [18] specify that an appropriate reference gene is an endogenous gene that exhibits relatively stable expression across a group of biological samples, including different cell or tissue types, different stages of development, or a range of experimental conditions.

*Setaria viridis* is an ideal genetic model to study C_4_ photosynthesis and cell wall biology [2, 3]. Several studies have attempted to validate the use of a number of ‘traditional’ housekeeper genes, including *UBIQUITIN*, *ACTIN*, *TUBULIN*, *GLYCERALDEHYDE 3*-*PHOSPHATE DEHYDROGENASE* and the ribosomal RNA, *18S*-*RNA*, to normalise gene expression data in *Setaria* tissues. However, the majority of these ‘traditional’ housekeeper genes returned poor expression stability under differing experimental conditions or across the samples tested, including seedlings, whole-stem, whole-leaf and a leaf developmental gradient [9, 22, 25]. Here, we assessed the suitability of eleven candidate reference genes for the accurate and reliable normalisation of gene expression quantification across thirteen developmentally distinct tissues of *S. viridis*, including internodes 4, 5 and 6, leaves 4, 5 and 6, inflorescence stems S1, S2 and S3, and the four developmental zones of the expanding internode, internode 5. Detailed expression information about the selected candidate reference genes is provided in Additional file 3: Table S2. Three candidate reference genes were selected from our existing RNA-Seq dataset that was generated from the four developmentally distinct zones of internode 5, an elongating internode [4]. The genes selected from this dataset included, *5*′-*ADENYLYLSULFATE REDUCTASE6* (*ASPR6*; *Sevir.3G358100*), *SERINE*-*THREONINE PROTEIN KINASE* (*STK*; *Sevir.1G021400*) and *ADIPOSE*-*REGULATORY PROTEIN* (*SEIPIN*)-*RELATED* (*SEIPIN; Sevir.2G298500*). Four additional candidate reference genes were next selected from a second *S. viridis* derived RNA-Seq dataset generated from an expanding leaf, leaf 3 [5], and included *DUAL SPECIFICITY PROTEIN PHOSPHATASE* (*DUSP*; *Sevir.4G179200*), *SERINE*-*THREONINE KINASE WNK1*-*RELATED* (*WNK1*; *Sevir.2G373600*), and one member each from the *GRAS DOMAIN* (*GRAS*; *Sevir.1G267700*) and *F*-*BOX DOMAIN* (*FBoxD*; *Sevir8G147200*) gene families. A further four candidate reference genes previously reported as appropriate candidates for *S. viridis* [9] or sorghum [14] gene expression normalisation were also included in this analysis, including *PROTEIN PHOSPHATASE2A* (*PP2A*; *Sevir.9G262700*), *PHOSPHOGLUCOMUTASE* (*PGM*; *Sevir.9G117100*), *CULLIN* (*CUL*; *Sevir.3G038900*) and *FOLYPOLYGLUTAMATE SYNTHASE* (*FPGS*; *Sevir.9G574400*).

### Assessment of the specificity and efficacy of selected candidate reference genes

**Fig. 1:**
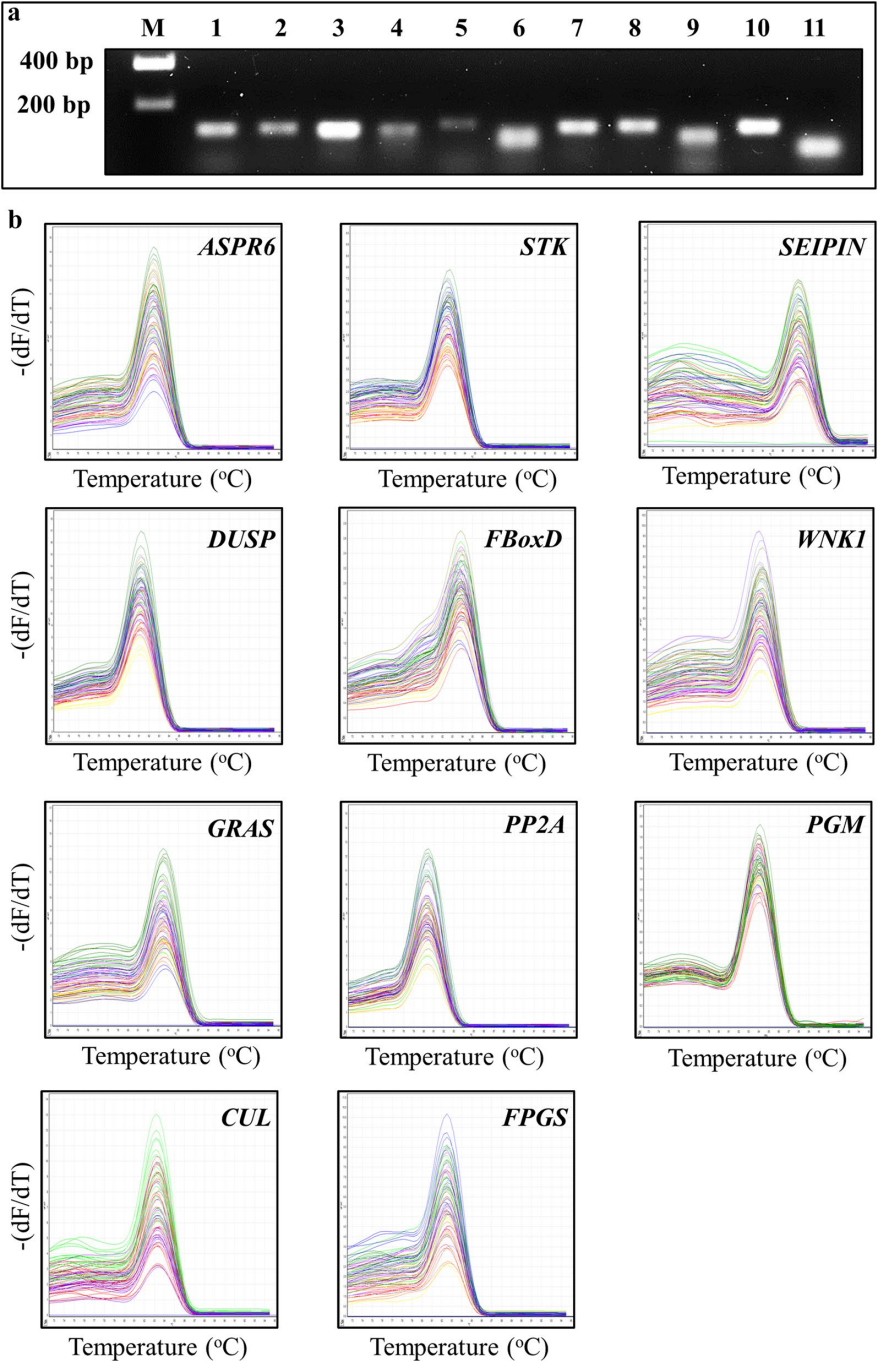
Specificity of each *S. viridis* candidate reference gene primer pair. **a** Agarose gel analysis of RT-PCR generated amplicons for each of the eleven assessed candidate reference genes. M, marker (200 base pair gene ruler); 1, *PP2A*; 2, *ASPR6*; 3, *PGM*; 4, *STK*; 5, *SEIPIN*; 6, *DUSP*; 7, *WNK1*; 8, *GRAS*; 9, *FBoxD*; 10, *CUL*, and; 11, *FPGS*. **b** Melt curve analysis of the eleven candidate reference genes assessed across thirteen developmentally distinct tissues of *S. viridis* tested (including, internodes 4, 5 and 6; leaves 4, 5 and 6; inflorescence stem stages S1, S2 and S3, and; the four developmental zones of the expanding internode, internode 5) showed a single peak for each primer pair at a specific annealing temperature. The -(dF/dT) value represents the raw fluorescence (F) versus temperature (T) values

Each pair of primers used for candidate reference gene analysis were designed via a combinatorial approach using the Primer3 ver4.0 (RID: nlx_156833) and NCBI Primer-BLAST (RRID: SCR_003095) online tools (see Additional file 2: Table S1). Following primer design, primer pairs of 11 candidate reference genes were tested using a standard RT-PCR approach and the construction of melt curves. For each primer pair, a single amplicon of the expected size was returned via RT-PCR analysis (Fig. 1a) of the cDNA template prepared from total RNA extracted from internode 5 as well as single melting curve peaks via RT-qPCR analysis across 52 tissue samples (Fig. 1b). The amplified products were next column purified and cloned into the pGEM^®^-T Easy cloning vector (Promega) in preparation for sequencing. Sequencing revealed that the desired region of each target transcript had been successfully amplified by RT-PCR with all eleven sequences returning 100% homology to the region targeted for amplification for each assessed candidate reference gene (see Additional file 4). The amplification efficiency (E), and the correlation coefficient value (R^2^), was also calculated from each standard curve, with E values ranging from 0.91 for *ASPR6*, to 1.09 for *CUL*, and R^2^ values ranging from 0.943 for *FBoxD* to 0.997 for *PP2A*, indicated that each primer pair was highly efficient and specific to the targeted region.

### Expression profiling of candidate reference genes

**Fig. 2:**
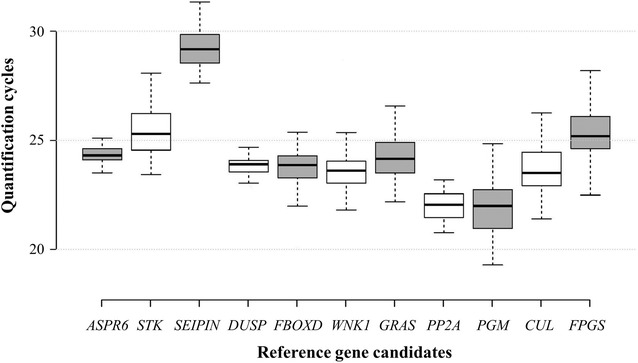
Reference gene quantification cycle (Cq) distributions. Considerable variability in Cq value was observed for the eleven candidate reference genes assessed across the internode, leaf and inflorescence tissue samples. The whisker caps show the distribution of the highest and lowest Cq values. The boxes indicate the first and third quartile, while the middle line marks the median. The majority of the assessed candidate reference genes returned an average Cq value ranging between 21.9 and 25.3 cycles

The box plot presented in Fig. 2 provides an overview of the expression level of the eleven candidate reference genes analysed. The RT-qPCR-derived quantification (Cq) values for these eleven candidates across all thirteen tissues assessed, was used for the generation of the data presented in Fig. 2. The mean Cq values varied from 19.2 for *PGM*, to 31.3 for *SEIPIN*, with a lower Cq value indicating a more abundant target transcript. The majority of the candidate reference genes analysed (10 out of 11), were determined to have an average Cq value that fell within the range of 21.9–25.3 cycles. Of these, *ASPR6* (Cq: 24.3) and *DUSP* (Cq: 23.9), returned the most compact Cq value distribution (i.e., exhibiting the least Cq value variance). This result strongly indicated that the expression of *ASPR6* and *DUSP* was the most stable of those assessed across the thirteen tissues sampled for analysis.

### Expression stability of candidate reference genes

**Fig. 3:**
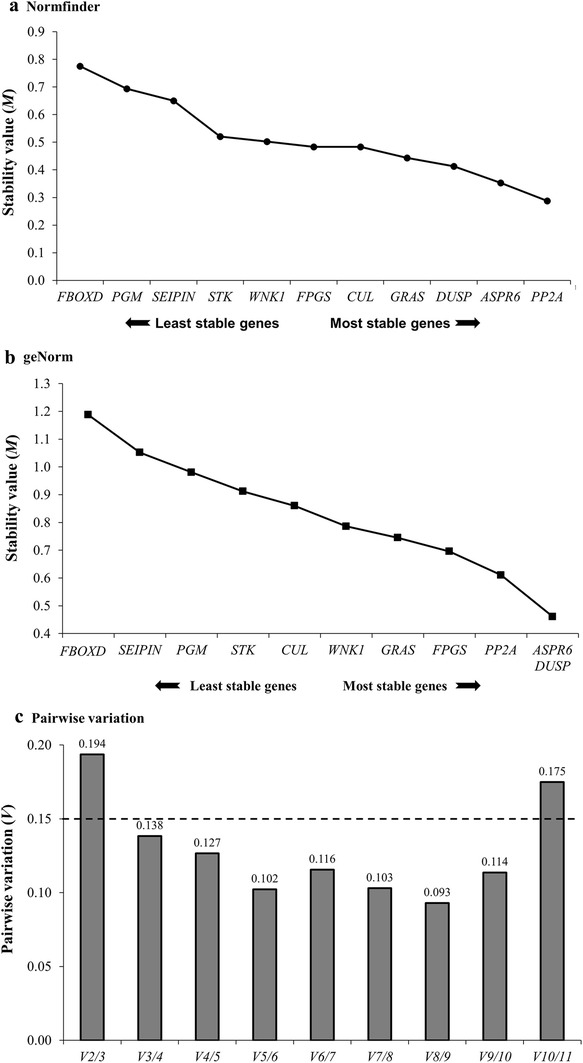
Average expression stability (*M*), ranking and pairwise variation (*V*_*n*_/*V*_(*n*+1)_) of the eleven reference gene candidates as calculated by NormFinder (**a**) and geNorm (**b**, **c**). A reduced value for ‘average expression stability’ indicates that the candidate reference gene is more stably expressed across the assessed tissues. Both the Normfinder (**a**) and the geNorm (**b**) approach ranked *PP2A*, *ASPR6* and *DUSP* as the most stably expressed of the eleven candidates analysed. **c** Pairwise variation (*V*_*n*_/*V*_(*n*+1)_) analysis by geNorm determines the optimal number of reference gene candidates required for the accurate and reliable normalisation of RT-qPCR generated data. The use of three reference genes to accurately and reliably normalise RT-qPCR generated data across *S. viridis* tissues was decided upon due to the *V*_3/4_ value being lower than the proposed threshold value of 0.15 [21]

To identify the most suitable reference genes, reference genes that exhibit stable expression across all assessed samples and/or experimental conditions, we next applied two frequently used statistical algorithms, NormFinder [20] and geNorm [21]. A stability value (*M*) was calculated and used to determine the optimal number of reference genes required for the accurate and reliable normalisation of RT-qPCR data across the different *S. viridis* tissues analysed. NormFinder is a Visual Basic tool that relies on a mathematical modelling approach to calculate the variation in gene expression between inter- (different tissues and developmental regions) and intra-related (biological replicates) groups, to rank the assessed candidates in order from least, to most stably expressed [22]. Specifically, an expressed gene that returns a low *M* value is classed as stably expressed, whereas a gene with a high *M* value is considered to have variable expression and therefore represents an unsuitable option for use as a reference gene to normalise RT-qPCR expression data [21]. NormFinder ranked *PP2A* (*M* value = 0.28), *ASPR6* (*M* = 0.35) and *DUSP* (*M* = 0.41), as the most stably expressed of the eleven assessed candidates, and therefore; the most suitable candidates for normalisation of *S. viridis* gene expression data (Fig. 3a). In previous studies, *PP2A* has also been identified as an ideal reference gene to normalise RT-qPCR data stemming from sorghum and pearl millet (*Pennisetum glaucum*) studies [14, 19]. Importantly, these previous studies assessed *PP2A* performance across various tissues (seedlings, leaves and roots), or under different conditions of abiotic stress (salt, cold, heat and drought) [14, 19]. *PP2A* has also been used previously to normalise gene expression data in both tobacco and *Arabidopsis thaliana* leaf samples, post viral infection [16, 23]. Following *PP2A*, *DUSP* and *ASPR6*, NormFinder ranked *GRAS* (*M* value = 0.44) as the next most suitable candidate to be used as a reference gene in *S. viridis* RT-qPCR analyses. In contrast, the remaining seven candidates returned *M* values ranging from 0.48 to 0.78, a result that indicates excessive variability in transcript abundance across the thirteen assessed tissues. These candidates were therefore considered unsuitable for use as normalisation controls in *S. viridis* RT-qPCR-based expression analyses.

Unlike the mathematical modelling approach used by NormFinder, geNorm evaluates reference gene suitability based on the stepwise exclusion of the least stable reference gene [21]. Although geNorm (Fig. 3b) utilises a different approach to that used by NormFinder (Fig. 3a), geNorm again identified *DUSP* and *ASPR6* as the most stably expressed of the eleven candidates assessed with both *DUSP* and *ASPR6* returning a *M* value score of 0.46. Further confidence in the NormFinder analysis was provided when the geNorm approach next ranked *PP2A*, with an *M* value of 0.61, as the third most suitable candidate. Therefore, both approaches identified the same three candidates as the most stably expressed, and thus, most suitable for use as reference genes to normalise *S. viridis* RT-qPCR data (Fig. 3). Similarly, and although not ranked in exactly the same order, *FBoxD*, *SEIPIN* and *PGM*, returned the highest *M* scores via geNorm analysis to indicate that these three candidates have variable expression across the assessed tissues and therefore, are not suitable for use as reference genes for *S. viridis* RT-qPCR data normalisation. Previous studies in *Setaria* and sorghum have demonstrated the requirement to use more than a single reference gene for the accurate and reliable normalisation of RT-qPCR data [9, 14, 22, 25]. GeNorm provides pairwise comparison (*V*_*n*_/*V*_*n*+1_) analysis of candidate reference genes for determination of the optimal number of reference genes required to ensure accurate and reliable RT-qPCR expression data normalisation [20]. The geNorm approach recommends that the threshold value is set to 0.15, below which the inclusion of an additional reference gene does not provide any further benefit to the analysis being performed [11, 20, 21]. Pairwise comparison analysis returned a *V*_2/3_ value of 0.194, above the recommended 0.15 threshold, to indicate that for *S. viridis* analyses, the use of two reference genes for RT-qPCR data normalisation would not be sufficient. Figure 3c further shows that a value of 0.138 was returned for the *V*_3/4_ pairwise comparison. Considering that the *V*_2/3_ value was above the threshold, and that the *V*_3/4_ value fell below the 0.15 threshold, the pairwise comparison analysis indicated that the optimal number of reference genes for accurate and reliable RT-qPCR data normalisation is three. The pairwise comparison analysis also clearly showed that the *V* value was significantly reduced via increasing the number of reference genes included in the analysis, for example; the use of six reference genes returned a *V*_8/9_ value of 0.093. However, the use of three reference genes for RT-qPCR data normalisation was considered the most practical choice. Taken together, the NormFinder and geNorm analyses clearly identified *PP2A*, *ASPR6* and *DUSP* as the best reference gene combination to ensure the accurate and reliable normalisation of RT-qPCR data generated from *S. viridis* tissues.

### Validation of selected candidate reference genes

**Fig. 4:**
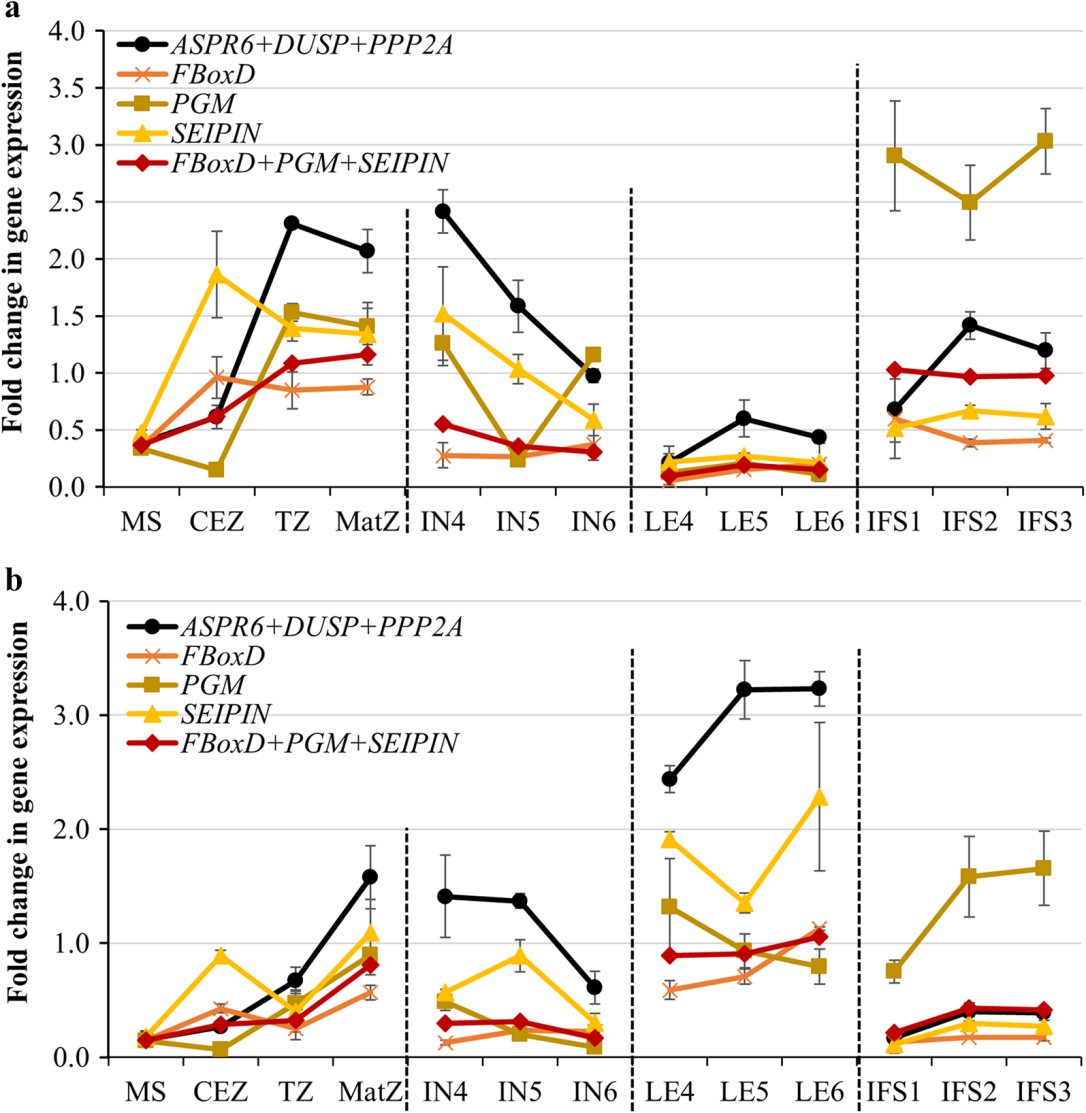
Profiling of *SvCAD2* and *SvCAD8* expression across thirteen developmentally distinct *Setaria viridis* tissues using the most suitable and least suitable sets of reference gene candidates. Normalised *SvCAD2* (**a**) and *SvCAD8* (**b**) expression using the most suitable set of reference genes (*ASPR6* + *DUSP* + *PP2A*) and the three least suitable reference genes individually (*FBoxD*, *PGM* and *SEIPIN*), and in combination with one another (*FBoxD* + *PGM* + *SEIPIN*). *MS* meristematic, *CEZ* cell expansion, TZ transitional, *MatZ* mature zones, *4–6IN* internode 4–6, *4–6LE* leaf 4–6, *IFS1–3* inflorescence stem stage 1–3

To validate the suitability of the three selected reference genes (*ASPR6*, *DUSP* and *PP2A*), and to demonstrate that *FBoxD*, *PGM* and *SEIPIN* (the three least suitably ranked candidates following NormFinder and geNorm analysis) are indeed the least suitable for RT-qPCR data normalisation, these two sets of putative candidate reference genes were used to generate an expression profile for two members of the *S. viridis CINNAMYL ALCOHOL DEHYDROGENASE* (*CAD*) gene family, namely *SvCAD2* (*Sevir.1G056800*) and *SvCAD8* (*Sevir.2G207500*), across whole internode samples (internode 4, 5 and 6), the whole leaf samples (leaf 4, 5 and 6), and the whole inflorescence stem samples (stages S1, S2 and S3). In addition, *SvCAD2* and *SvCAD8* expression was also quantified across the four developmentally distinct regions of elongating internode 5 using these two sets of candidate reference genes. In the lignin biosynthesis pathway, CAD proteins are one of the eleven key enzymes that function as dimeric alcohol dehydrogenases to catalyse the reduction of the cinnamylaldehydes, coumaraldehyde, synapaldehyde and coniferaldehyde, to produce the lignin precursors, coumaryl alcohol, synapyl alcohol and coniferyl alcohol, respectively [26]. When applying the three best reference gene candidates (*ASPR6*, *DUSP* and *PP2A*) for expression normalisation, RT-qPCR revealed that *SvCAD2* and *SvCAD8* transcript abundance increased sharply in internode 5 (an elongating internode) and internode 4 (a mature internode) as well as in inflorescence S2 (an expanding inflorescence) and inflorescence S3 (a mature inflorescence; Fig. 4). A similar expression profile for *SvCAD2* and *SvCAD8* was also observed across the four developmentally distinct zones of internode 5, that is: *SvCAD2* and *SvCAD8* transcript abundance slowly increased from the MS to the CEZ zone (zones composed of immature and elongating tissues) before sharply increasing in the TZ zone (elongating and mature tissues) and peaking in the MatZ zone (mature tissues). Further, the *SvCAD2* and *SvCAD8* expression profiles across the four developmental zones of internode 5 generated here via RT-qPCR, correlated strongly with those obtained in our previous RNA-Seq dataset (see Additional file 5: Figure S2), a dataset profiling gene expression across the same four zones of elongating internode 5 [4]. Interestingly, when the *ASPR6*, *DUSP* and *PP2A* reference gene combination was used to normalise *SvCAD2* and *SvCAD8* expression across leaves 4, 5 and 6, an opposing expression profile was observed. More specifically, RT-qPCR revealed *SvCAD2* and *SvCAD8* to be expressed at a low level in leaf 4, a mature leaf, more abundantly expressed in elongating leaf 5, and highly expressed in leaf 6, a young and developing leaf. RT-qPCR also revealed a clear difference in transcript abundance of *SvCAD2* and *SvCAD8* amongst the internode, leaf and inflorescence stem samples. Namely, the *SvCAD2* transcript was highly abundant in the internode and inflorescence samples compared to the leaf samples, whereas *SvCAD8* was predominantly expressed in the three leaf samples. Abundant expression of the *SvCAD2* transcript in the internode samples was not surprising, as Saballos et al. [27] have previously demonstrated that the orthologous protein in sorghum, *Sb*CAD2, primarily functions to direct lignification of stem tissues during flowering. Our expression data for *SvCAD2* infers a similar, stem-specific function in the lignin biosynthesis pathway for CAD2 in the *S. viridis* inflorescence.

The candidate reference genes, *FBoxD*, *PGM* and *SEIPIN*, ranked by NormFinder and geNorm as the least stably expressed and therefore, the least suitable for use as reference genes for RT-qPCR normalisation were also included in this analysis. Interestingly, when these three unsuitable reference gene candidates were used to normalise RT-qPCR data to profile *SvCAD2* and *SvCAD8* expression, the expression of these two *CAD*s was determined to be highly variable across the assessed tissues. Furthermore, when *FBoxD*, *PGM* and *SEIPIN* were used to normalise *SvCAD2* and *SvCAD8* expression, the expression of these two *CAD*s was repeatedly underestimated, or even obscured, in each of the analysed tissues. Similar results were obtained when the expression of the other four members of *S. viridis CAD* gene family, *SvCAD3*, *SvCAD5*, *SvCAD6* and *SvCAD7*, were also normalised with the three reference genes determined least suitable (see Additional file 6: Figure S3). Taken together, the RT-qPCR profiling of *CAD* gene expression strongly supported the NormFinder and geNorm analyses identifying *ASPR6*, *DUSP* and *PP2A* as the most appropriate set of reference genes for the accurate and reliable gene expression normalisation in future *S. viridis* RT-qPCR-based studies.

## Conclusion

In conclusion, this study provides a list of novel reference genes for the accurate and reliable normalisation of gene expression data generated by RT-qPCR analyses of the model C_4_ plant, *Setaria viridis*. Here we identified *ASPR6*, *DUSP* and *PP2A* as the most stably expressed, and therefore most suitable set of reference genes for the normalisation of RT-qPCR generated gene expression data across *S. viridis* tissues. Furthermore, we demonstrated the combined performance of the three selected reference genes for the accurate and reliable normalisation of *CAD* gene expression across developmentally distinct *S. viridis* tissues. It would however behove researchers to experimentally validate the stability of the reference genes identified in this investigation for their own experimental treatments and purposes. The plant material and organs sampled in this study were taken within a restricted period of the day when the plants were actively photosynthesising. Therefore, the suitability of the three reference genes identified here as ideal candidates for RT-qPCR data normalisation, would require empirical validation prior to use as reference genes for an investigation of circadian rhythms for example. The findings presented in this study form a highly useful platform for future profiling of the expression of genes of interest in the *S. viridis*, findings that can also be potentially directly transferred to other closely related and agronomically important C_4_ crop species.

## Additional files

**Additional file 1: Figure S1.** *Setaria viridis* tissues sampled for RT-qPCR analysis. *Setaria viridis* internode and leaf samples were harvested at the 50% ear-emergence stage. **A** Sampling of internode 5 via the division of this elongating internode into four samples representing enriched samples of the developmentally distinct zones, the meristematic (MS), cell expansion (CEZ), transitional (TZ) and maturation (MatZ) zones. **B** Division of the primary tiller into internodes 1–6 (nodes depicted by discontinuous lines), and numbered acropetally from mature to younger tissues. Whole internodes, internode 4, 5 and 6, were sampled to represent enriched samples of mature, transitioning, and elongating tissues, respectively. **C** Leaves on the primary tiller, which attach to the base of each internode, were numbered from 2 to 6 according to the number of the internode from which leaf was detached, with leaf numbers 4, 5 and 6 sampled to represent enriched samples of mature, transitioning and elongating tissues, respectively. **D** Inflorescence stem samples were harvested at 3 distinct stages including the 50% ear emergence (S1; 20–25 DAG), flowering (S2; 27–32 DAG) and milky dough (S3; 40–45 DAG) stages to represent enriched samples of elongating, transitioning and mature tissues, respectively.

**Additional file 2: Table S1.** Table of reference gene candidates and the primers used for subsequent RT-qPCR analyses.

**Additional file 3: Table S2.** Selection of reference gene candidates for normalisation of *S. viridis* RT-qPCR data. Seven reference gene candidates (*ASPR6*, *STK*, *SEIPIN*, *DUSP*, *FBoxD*, *WNK1 *and *GRAS*) were selected based on two RNA-seq datasets, the elongating internode 5 and the leaf 3. The internode and leaf reference gene candidates exhibited stable transcript levels across 4 regions of the internode 5 and leaf 3, respectively.* PP2A*, *PGM*, *CUL* and *FPGS* were 4 potential reference genes identified from previous publications.

**Additional file 4.** Confirmation of gene specificity of RT-qPCR primer pairs by sequencing of RT-PCR cloned into the pGEMT-Easy cloning vector. Each chromatograph shows the sequence of the amplified product of the eleven assessed primer pairs was a 100% match to the targeted region of the transcript of each reference gene candidate.

**Additional file 5: Figure S2.** Analysis of *Setaria viridis* *CAD* gene expression according to the RNA-Seq dataset published in [4]. The transcript abundance of each of the detected members of the *S. viridis* *CAD* gene family across the four developmentally distinct zones of the elongating internode, internode 5, according to the dataset published by Martin et al. [4]. Of the six *CAD*s identified from the dataset, the* SvCAD2* (*Sevir.1G056800*) transcript was determined to be the most abundance in internode 5 and further, *SvCAD2* returned an expression profile expected of a gene that encodes a protein that plays a functional role in the formation of secondary cell walls, that is; less abundant in young or undifferentiated tissues and with a greatly enhanced abundance in transitioning and/or mature tissues.

**Additional file 6: Figure S3.** Profiling of the expression of *SvCAD3*, *SvCAD5*, *SvCAD6* and *SvCAD7* following normalisation with the most suitable, and the least suitable, set of reference genes. **A**–**D** RT-qPCR data to profile the expression of* SvCADs*, *SvCAD3* (**A**; *Sevir.6G025000*),* SvCAD5* (**B**; *Sevir6G024400*), *SvCAD6* (**C**; *Sevir7G014100*), and *SvCAD7* (**D**; *Sevir7G245600*) was normalised using the set of the three most suitable reference genes (*ASPR6*, *DUSP* and *PP2A*) and with the set of the three least suitable reference genes (*FBoxD*,* PGM* and *SEIPIN*).

## Abbreviations

ASPR6: adenylylsulfate reductase 6
CAD: cinnamyl alcohol dehydrogenase
CEZ: cell expansion zone
CUL: cullin
DUSP: dual specificity protein phosphatase
FBoxD: F-box domain
FPGS: folylpolyglutamate synthase
GRAS: GRAS domain family
MS: meristematic zone; MatZ: mature zone
PGM: phosphoglucomutase
PP2A: serine/theronine-protein phosphatase 2A
SEIPIN: adipose-regulatory protein (SEIPIN)-related
STK: serine–threonine protein kinase
TZ: transitional zone
WNK1: serine/theronine-protein kinase WNK1-related
