# Supplementary material for: Reference gene identification for reliable normalisation of quantitative RT-PCR data in Setaria viridis
Source: Plant Methods. 2018 Mar 21;14:24. doi: 10.1186/s13007-018-0293-8 (PMC5861610; doi:10.1186/s13007-018-0293-8)
Supplement: Supplementary file 1 — Additional file 1: Figure S1. Setaria viridis tissues sampled for RT-qPCR analysis. Setaria viridis internode and leaf samples were harvested at the 50% ear-emergence stage. A Sampling of internode 5 via the division of this elongating internode into four samples representing enriched samples of the developmentally distinct zones, the meristematic (MS), cell expansion (CEZ), transitional (TZ) and maturation (MatZ) zones. B Division of the primary tiller into internodes 1–6 (nodes depicted by discontinuous lines), and numbered acropetally from mature to younger tissues. Whole internodes, internode 4, 5 and 6, were sampled to represent enriched samples of mature, transitioning, and elongating tissues, respectively. C Leaves on the primary tiller, which attach to the base of each internode, were numbered from 2 to 6 according to the number of the internode from which leaf was detached, with leaf numbers 4, 5 and 6 sampled to represent enriched samples of mature, transitioning and elongating tissues, respectively. D Inflorescence stem samples were harvested at 3 distinct stages including the 50% ear emergence (S1; 20–25 DAG), flowering (S2; 27–32 DAG) and milky dough (S3; 40–45 DAG) stages to represent enriched samples of elongating, transitioning and mature tissues, respectively. [file 13007_2018_293_MOESM1_ESM.docx]

Additional file 1

Reference gene identification for reliable normalisation of quantitative RT-PCR data in *Setaria viridis*

Duc Quan Nguyen^1^, Andrew L. Eamens^1†^ and Christopher P. L. Grof^1*†^

^1^ Centre for Plant Science, School of Environmental and Life Sciences, University of Newcastle, University Drive, Callaghan, NSW 2308, Australia

*** Correspondence:**Christopher Grof
[chris.grof@newcastle.edu.au](mailto:chris.grof@newcastle.edu.au)

^†^ These authors contributed equally to this work


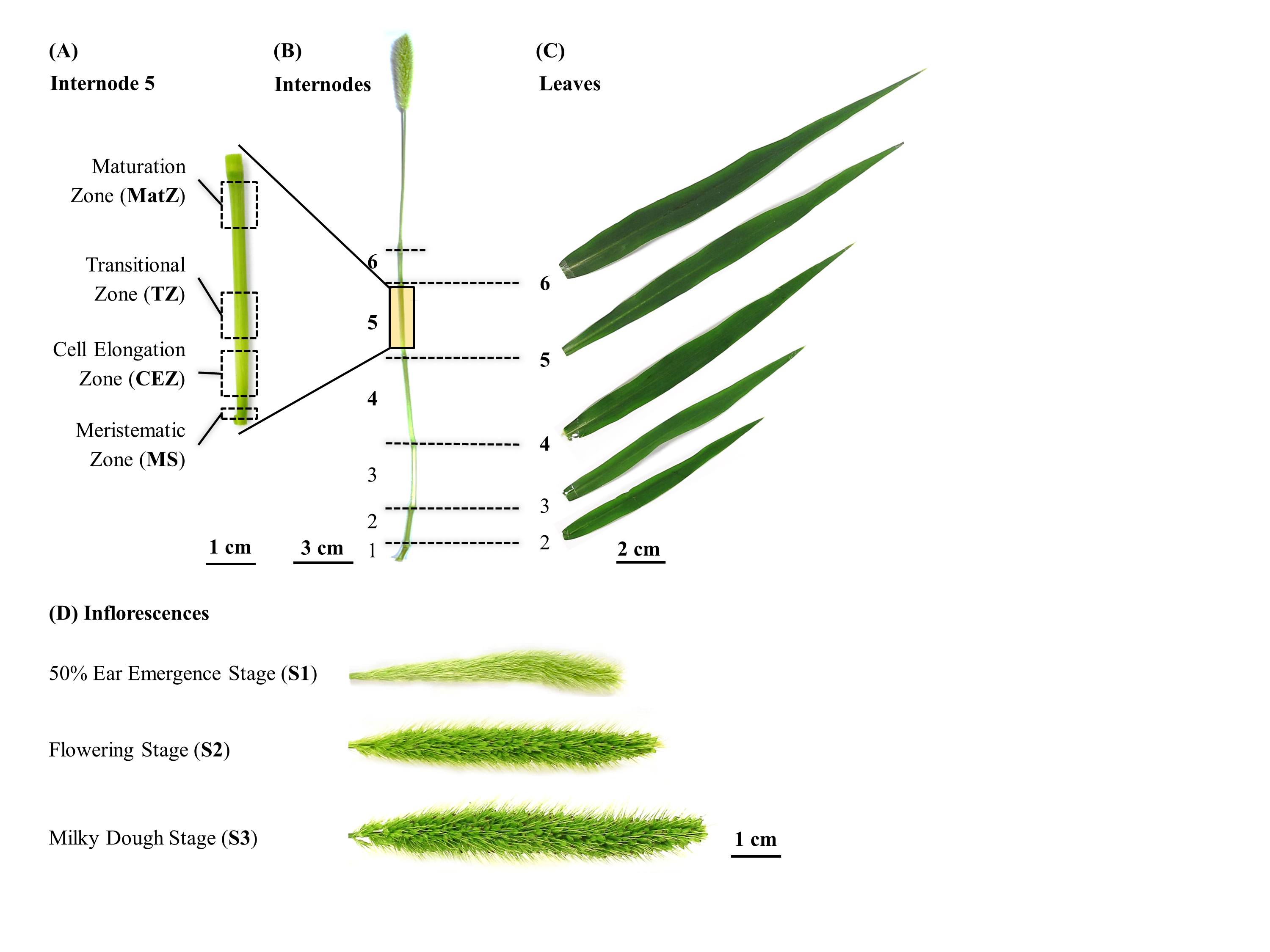


**Figure S1:** ***Setaria viridis* tissues sampled for RT-qPCR analysis.** *Setaria viridis* internode and leaf samples were harvested at the 50% ear-emergence stage (20-25 days after germination; DAG). **(A)** Sampling of internode 5 via the division of this elongating internode into four samples representing enriched samples of the developmentally distinct zones, the meristematic (**MS**), cell expansion (**CEZ**), transition (**TZ**) and mature (**MatZ**) zones. **(B)** Division of the primary tiller into internodes 1 to 6 (nodes depicted by discontinuous lines), and numbered acropetally from mature to younger tissues. Whole internodes, internode 4, 5 and 6, were sampled to represent enriched samples of mature, transitioning, and elongating tissues, respectively. **(C)** Leaves on the primary tiller, which attach to the base of each internode, were numbered from 2 to 6 according to the number of the internode from which leaf was detached, with leaf numbers 4, 5 and 6 sampled to represent enriched samples of mature, transitioning and elongating tissues, respectively. (**D**) Inflorescence stem samples were harvested at 3 distinct stages, including the 50% ear emergence (S1; 20-25 DAG), flowering (S2; 27-32 DAG) and milky dough (S3; 40-45 DAG) stages to represent enriched samples of elongating, transitioning and mature tissues, respectively.
