# Supplementary material for: Reference gene identification for reliable normalisation of quantitative RT-PCR data in Setaria viridis
Source: Plant Methods. 2018 Mar 21;14:24. doi: 10.1186/s13007-018-0293-8 (PMC5861610; doi:10.1186/s13007-018-0293-8)
Supplement: Supplementary file 2 — Additional file 2: Table S1. Table of reference gene candidates and the primers used for subsequent RT-qPCR analyses. [file 13007_2018_293_MOESM2_ESM.docx]

Additional file 2

Reference gene identification for reliable normalisation of quantitative RT-PCR data in *Setaria viridis*

Duc Quan Nguyen^1^, Andrew L. Eamens^1†^ and Christopher P. L. Grof^1*†^

^1^ Centre for Plant Science, School of Environmental and Life Sciences, University of Newcastle, University Drive, Callaghan, NSW 2308, Australia

*** Correspondence:**Christopher Grof
[chris.grof@newcastle.edu.au](mailto:chris.grof@newcastle.edu.au)

^†^ These authors contributed equally to this work

**Table S1: Reference gene candidates and the primers used for subsequent RT-qPCR analyses**

|  | **No.** | **Gene acronyms** | **Accession no.** | **Primers** | **Amplicon  length (bp)** |
| --- | --- | --- | --- | --- | --- |
| **Internode candidates** | 1 | ***ASPR6*** | *Sevir.3G358100* | F: ATGGTGCCCTTTCTCACAAC R: GCATGACGTTGGACTCTTCA | 101 |
|  | 2 | ***STK*** | *Sevir.1G021400* | F: CCCCATGGATATGACGATTC R: ATTGTCCTGGAAAACGCATC | 97 |
|  | 3 | ***SEIPIN*** | *Sevir.2G298500* | F: AGGTGACCGAGACCCTGAAT R: AGCGTCAGCGATACCTCAAG | 115 |
| **Leaf**  **candidates** | 4 | ***DUSP*** | *Sevir.4G179200* | F: CTGAAAGAGCTGGCGAAGTG R: GCTGTCCATCATGCTATCGC | 86 |
|  | 5 | ***FBoxD*** | *Sevir.8G147200* | F: GGTTGTGGCAGGAGGACTAC R: GAGCTCTCTGTGCCTCCATC | 79 |
|  | 6 | ***WNK1*** | *Sevir.2G373600* | F: GTTGTCAATGTGCGGAGCTA R: TCCATCATCAGAGCTTCCTG | 104 |
|  | 7 | ***GRAS*** | *Sevir.1G267700* | F: ACCTTGGAATCCCCTTTGAG R: ACCAACAGGGAGGACAACAG | 110 |
| **From**  **previous studies** | 8 | ***PP2A*** | *Sevir.9G262700* | F: ATGTGACACGGAGAACACCA R: TGTTTCTGACCAGCAACCAC | 96 |
|  | 9 | ***PGM*** | *Sevir.9G117100* | F: AGAGTTGGTTGAACGGATGG R: ATGTTTCGGTCAGCATCTCC | 96 |
|  | 10 | ***CUL*** | *Sevir.3G038900* | F: TGTTTCCAGGGCCATACACTC R: CGCAGAAAGTAGCCAGCAATT | 112 |
|  | 11 | ***FPGS*** | *Sevir.9G574400* | F: GAAGCAGGGACACAAGGACA R: ACAATCTGTGCTCGGCCTT | 108 |
|  | | | | | |
