# Supplementary material for: Reference gene identification for reliable normalisation of quantitative RT-PCR data in Setaria viridis
Source: Plant Methods. 2018 Mar 21;14:24. doi: 10.1186/s13007-018-0293-8 (PMC5861610; doi:10.1186/s13007-018-0293-8)
Supplement: Supplementary file 3 — Additional file 3: Table S2. Selection of reference gene candidates for normalisation of S. viridis RT-qPCR data. Seven reference gene candidates (ASPR6, STK, SEIPIN, DUSP, FBoxD, WNK1 and GRAS) were selected based on two RNA-seq datasets, the elongating internode 5 and the leaf 3. The internode and leaf reference gene candidates exhibited stable transcript levels across 4 regions of the internode 5 and leaf 3, respectively. PP2A, PGM, CUL and FPGS were 4 potential reference genes identified from previous publications. [file 13007_2018_293_MOESM3_ESM.docx]

Additional file 3

Reference gene identification for reliable normalisation of quantitative RT-PCR data in *Setaria viridis*

Duc Quan Nguyen^1^, Andrew L. Eamens^1†^ and Christopher P. L. Grof^1*†^

^1^ Centre for Plant Science, School of Environmental and Life Sciences, University of Newcastle, University Drive, Callaghan, NSW 2308, Australia

*** Correspondence:**Christopher Grof
[chris.grof@newcastle.edu.au](mailto:chris.grof@newcastle.edu.au)

^†^ These authors contributed equally to this work

**Table S2:** **Selection of reference gene candidates for normalisation of *S. viridis* RT-qPCR data.** Seven reference gene candidates (*ASPR6*, *STK*, *SEIPIN*, *DUSP*, *FboxD*, *WNK1* and *GRAS*) were selected based on analysis of FPKM and fold change values from two RNA-seq datasets, the elongating internode 5 and the leaf 3 [1, 2]. The internode and leaf reference gene candidates exhibited stable transcript levels across 4 regions of the internode 5 and leaf 3, respectively. *PP2A*, *PGM*, *CUL* and *FPGS* were 4 potential reference genes identified from previous publications [3, 4].

|  | **Gene acronym** | **Accession no.** | **Gene name** | **MS** | **CEZ** | **TZ** | **MatZ** | **LSec1** | **LSec2** | **LSec3** | **LSec4** |
| --- | --- | --- | --- | --- | --- | --- | --- | --- | --- | --- | --- |
| **Internode**  **candidates** | ***ASPR6*** | *Sevir.3G358100* | *Adenylylsulfate reductase 6* | 17.3 | 18.2 | 17.9 | 20.0 | 13.3 | 5.5 | 3.6 | 3.3 |
|  | ***STK*** | *Sevir.1G021400* | *Serine-Threonine protein kinase* | 16.0 | 15.0 | 14.0 | 14.6 | 17.6 | 13.2 | 11.0 | 10.4 |
|  | ***SEIPIN*** | *Sevir.2G298500* | *Adipose-regulatory protein (SEIPIN)-Related* | 3.4 | 3.1 | 3.4 | 3.3 | 3.3 | 3.0 | 3.9 | 4.5 |
| **Leaf**  **candidates** | ***DUSP*** | *Sevir.4G179200* | *Dual specificity protein phosphatase* | 23.2 | 21.8 | 16.4 | 17.7 | 17.9 | 18.7 | 19.8 | 18.1 |
|  | ***FBoxD*** | *Sevir.8G147200* | *F-box domain* | 16.1 | 12.3 | 13.3 | 17.6 | 19.0 | 20.9 | 18.5 | 19.0 |
|  | ***WNK1*** | *Sevir.2G373600* | *Serine/Theronine-protein kinase WNK1-Related* | 35.5 | 44.4 | 34.0 | 42.2 | 47.8 | 52.6 | 53.1 | 48.6 |
|  | ***GRAS*** | *Sevir.1G267700* | *GRAS domain family* | 21.5 | 23.1 | 26.6 | 20.0 | 34.7 | 37.6 | 34.6 | 37.4 |
| **From**  **previous studies** | ***PP2A*** | *Sevir.9G262700* | *Serine/Theronine-protein phosphatase 2A* | 62.8 | 58.5 | 39.8 | 38.2 | 47.1 | 32.7 | 59.0 | 71.5 |
|  | ***PGM*** | *Sevir.9G117100* | *Phosphoglucomutase* | 251.8 | 502.1 | 373.3 | 185.6 | 285.6 | 255.2 | 345.4 | 351.4 |
|  | ***CUL*** | *Sevir.3G038900* | *Cullin* | 97.5 | 80.4 | 67.3 | 74.2 | 62.7 | 27.5 | 29.8 | 30.7 |
|  | ***FPGS*** | *Sevir.9G574400* | *Folylpolyglutamate synthase* | 31.5 | 20.1 | 26.5 | 34.6 | 15.1 | 11.7 | 9.4 | 8.0 |
|  | | | | | | | | | | | |

1. Martin AP, Palmer WM, Brown C, Abel C, Lunn JE, Furbank RT, et al. A developing *Setaria viridis* internode: an experimental system for the study of biomass generation in a C_4_ model species. Biotechnol Biofuels. 2016;9:45-57. doi: 10.1186/s13068-016-0457-6.

2. Studer AJ, Schnable JC, Weissmann S, Kolbe AR, McKain MR, Shao Y, et al. The draft genome of the C(3) panicoid grass species *Dichanthelium oligosanthes*. Genome Biol. 2016;17:223-41. doi: 10.1186/s13059-016-1080-3.

3. Lambret-Frotte J, de Almeida LC, de Moura SM, Souza FL, Linhares FS, Alves-Ferreira M. Validating internal control genes for the accurate normalization of qPCR expression analysis of the novel model plant *Setaria viridis*. PloS one. 2015;10:1-22. doi: 10.1371/journal.pone.0135006.

4. Reddy S, Srinivas Reddy D, Sivasakthi K, Bhatnagar-Mathur P, Vadez V, Sharma KK. Evaluation of sorghum [*Sorghum bicolor* (L.)] reference genes in various tissues and under abiotic stress conditions for quantitative real-time PCR data normalization. Front Plant Sci. 2016;7:529-43. doi: 10.3389/fpls.2016.00529.
