# Supplementary material for: Reference gene identification for reliable normalisation of quantitative RT-PCR data in Setaria viridis
Source: Plant Methods. 2018 Mar 21;14:24. doi: 10.1186/s13007-018-0293-8 (PMC5861610; doi:10.1186/s13007-018-0293-8)

Additional file 4

Reference gene identification for reliable normalisation of quantitative RT-PCR data in *Setaria viridis*

Duc Quan Nguyen^1^, Andrew L. Eamens^1†^ and Christopher P. L. Grof^1*†^

^1^ Centre for Plant Science, School of Environmental and Life Sciences, University of Newcastle, University Drive, Callaghan, NSW 2308, Australia

*** Correspondence:**Christopher Grof
[chris.grof@newcastle.edu.au](mailto:chris.grof@newcastle.edu.au)

^†^ These authors contributed equally to this work

**Supplementary Data 1: Confirmation of gene specificity of RT-qPCR primer pairs by sequencing of RT-PCR cloned into** **the pGEMT-Easy cloning vector.** Each chromatograph shows the sequence of the amplified product of the eleven assessed primer pairs was a 100% match to the targeted region of the transcript of each reference gene candidate.


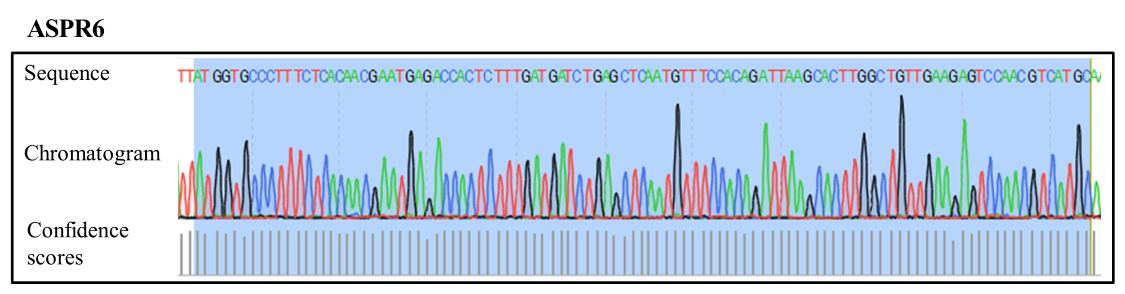


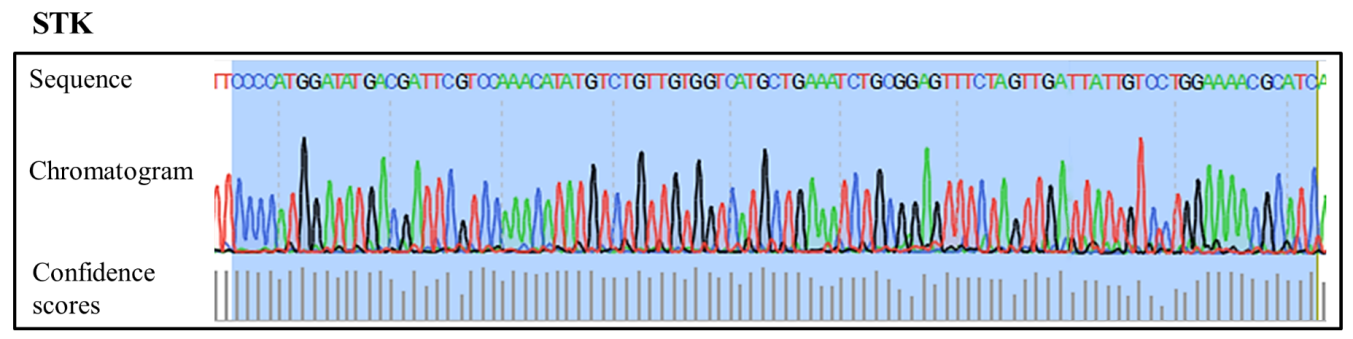


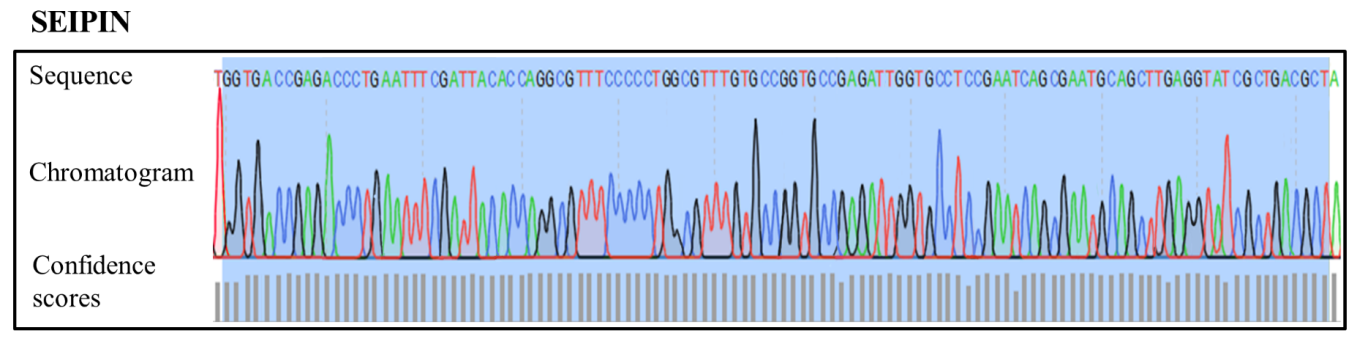


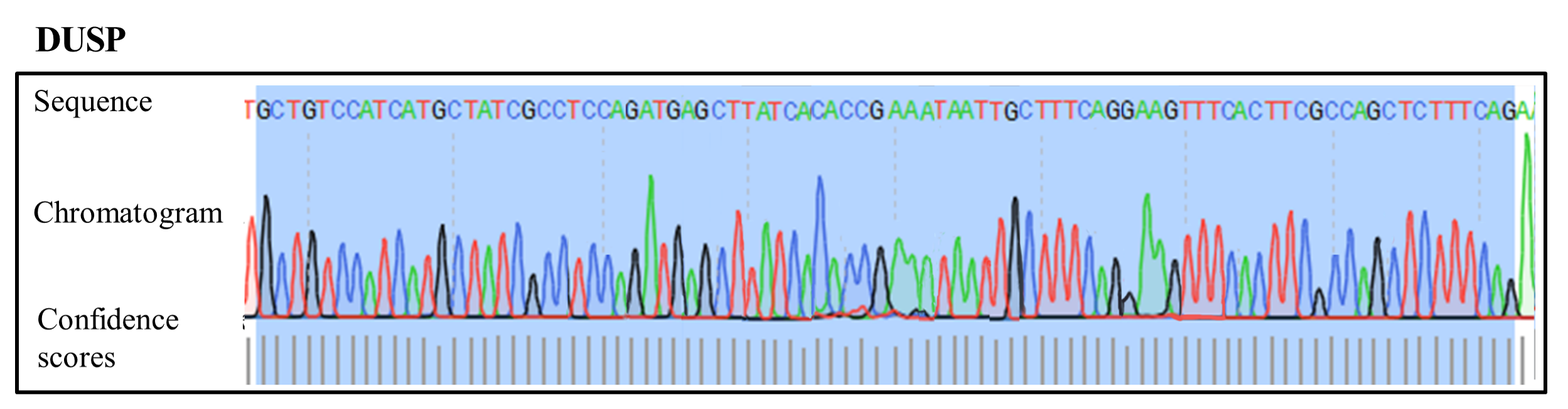


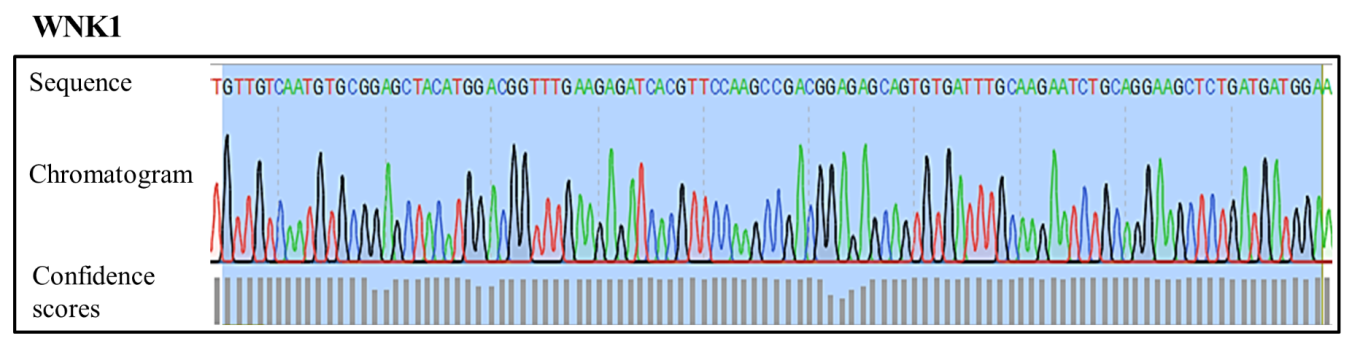


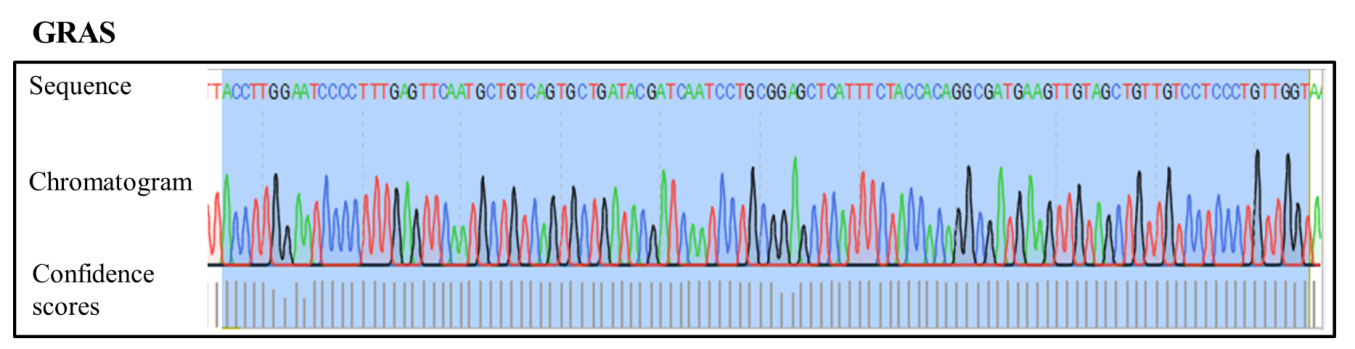


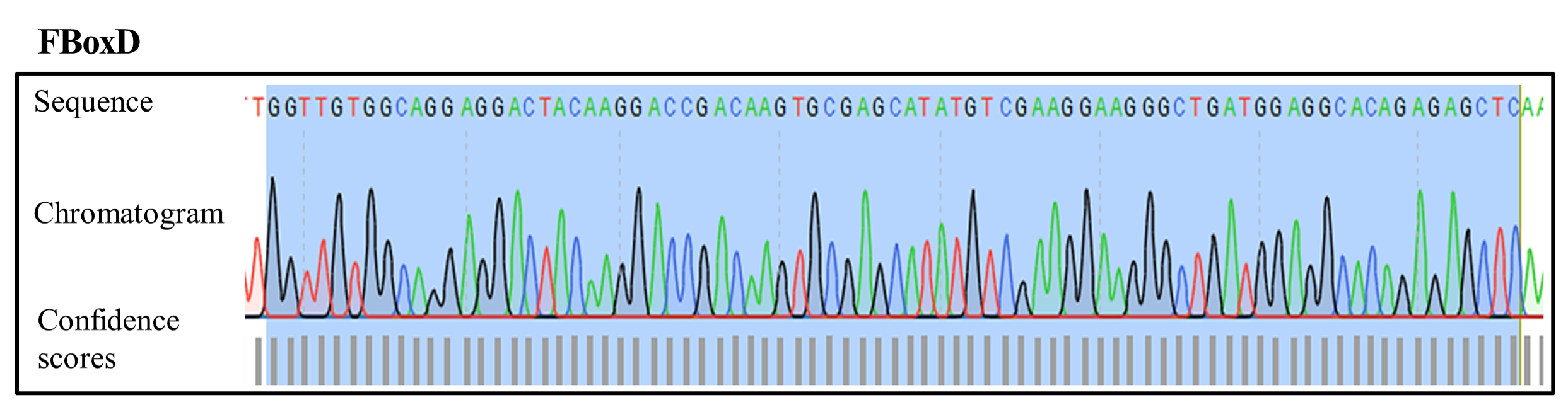


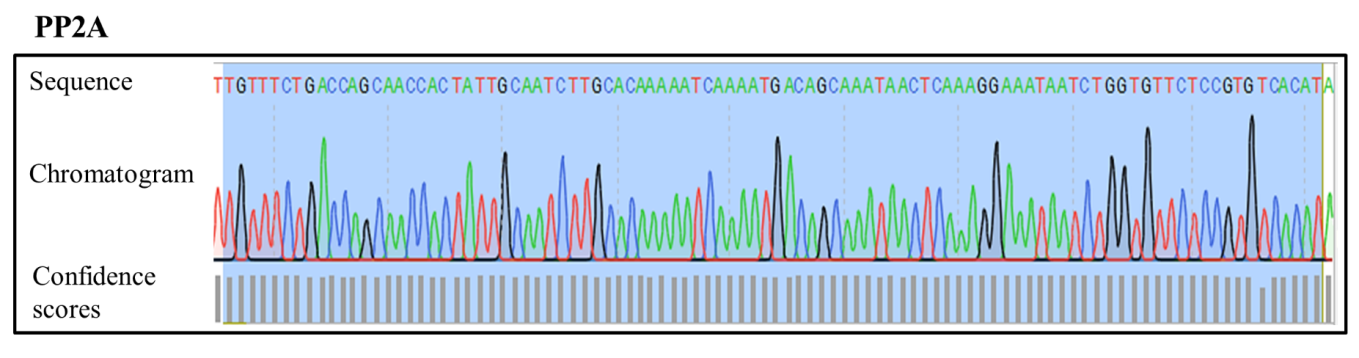


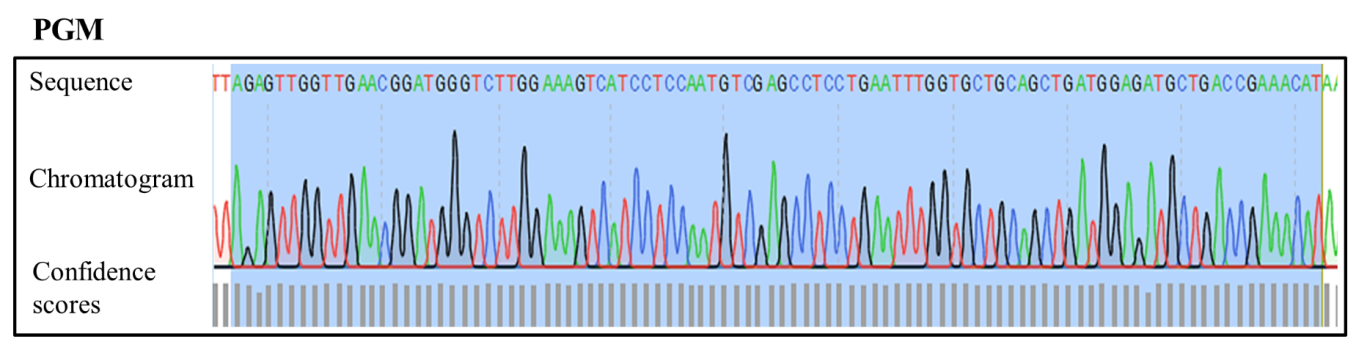


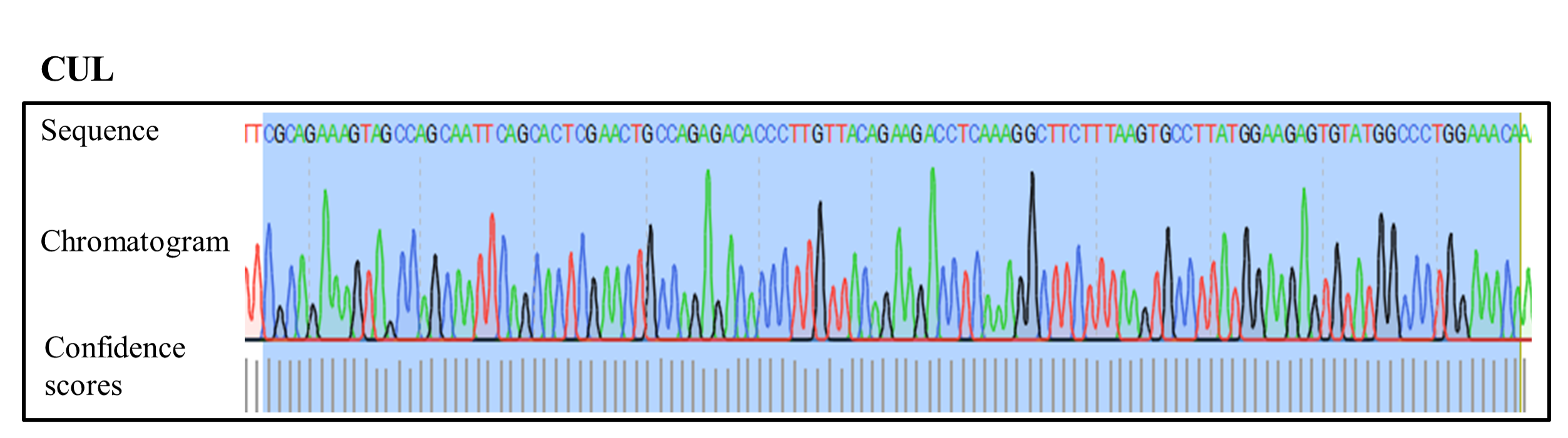


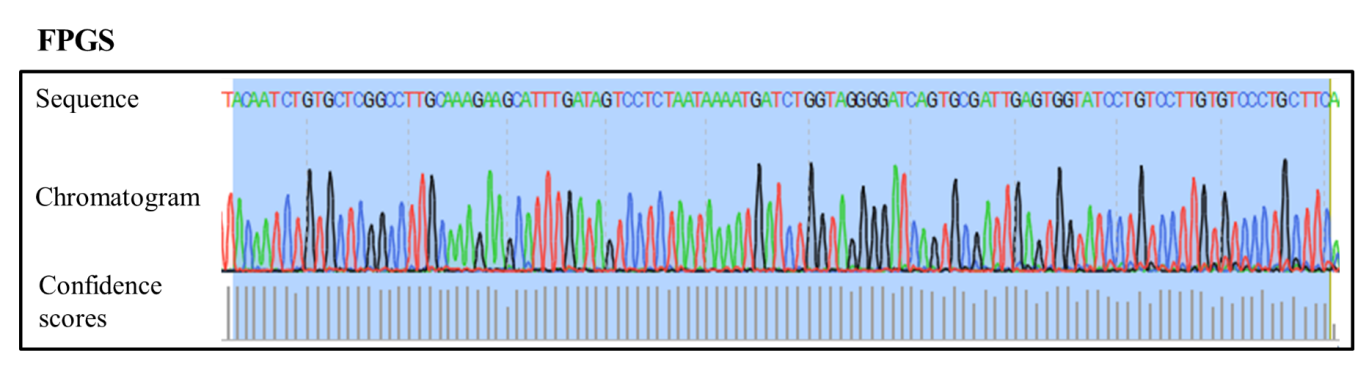

Supplement: Supplementary file 4 — Additional file 4. Confirmation of gene specificity of RT-qPCR primer pairs by sequencing of RT-PCR cloned into the pGEMT-Easy cloning vector. Each chromatograph shows the sequence of the amplified product of the eleven assessed primer pairs was a 100% match to the targeted region of the transcript of each reference gene candidate. [file 13007_2018_293_MOESM4_ESM.docx]
