# Supplementary material for: Reference gene identification for reliable normalisation of quantitative RT-PCR data in Setaria viridis
Source: Plant Methods. 2018 Mar 21;14:24. doi: 10.1186/s13007-018-0293-8 (PMC5861610; doi:10.1186/s13007-018-0293-8)
Supplement: Supplementary file 5 — Additional file 5: Figure S2. Analysis of Setaria viridis CAD gene expression according to the RNA-Seq dataset published in [4]. The transcript abundance of each of the detected members of the S. viridis CAD gene family across the four developmentally distinct zones of the elongating internode, internode 5, according to the dataset published by Martin et al. [4]. Of the six CADs identified from the dataset, the SvCAD2 (Sevir.1G056800) transcript was determined to be the most abundance in internode 5 and further, SvCAD2 returned an expression profile expected of a gene that encodes a protein that plays a functional role in the formation of secondary cell walls, that is; less abundant in young or undifferentiated tissues and with a greatly enhanced abundance in transitioning and/or mature tissues. [file 13007_2018_293_MOESM5_ESM.docx]

Additional file 5

Reference gene identification for reliable normalisation of quantitative RT-PCR data in *Setaria viridis*

Duc Quan Nguyen^1^, Andrew L. Eamens^1†^ and Christopher P. L. Grof^1*†^

^1^ Centre for Plant Science, School of Environmental and Life Sciences, University of Newcastle, University Drive, Callaghan, NSW 2308, Australia

*** Correspondence:**Christopher Grof
[chris.grof@newcastle.edu.au](mailto:chris.grof@newcastle.edu.au)

^†^ These authors contributed equally to this work


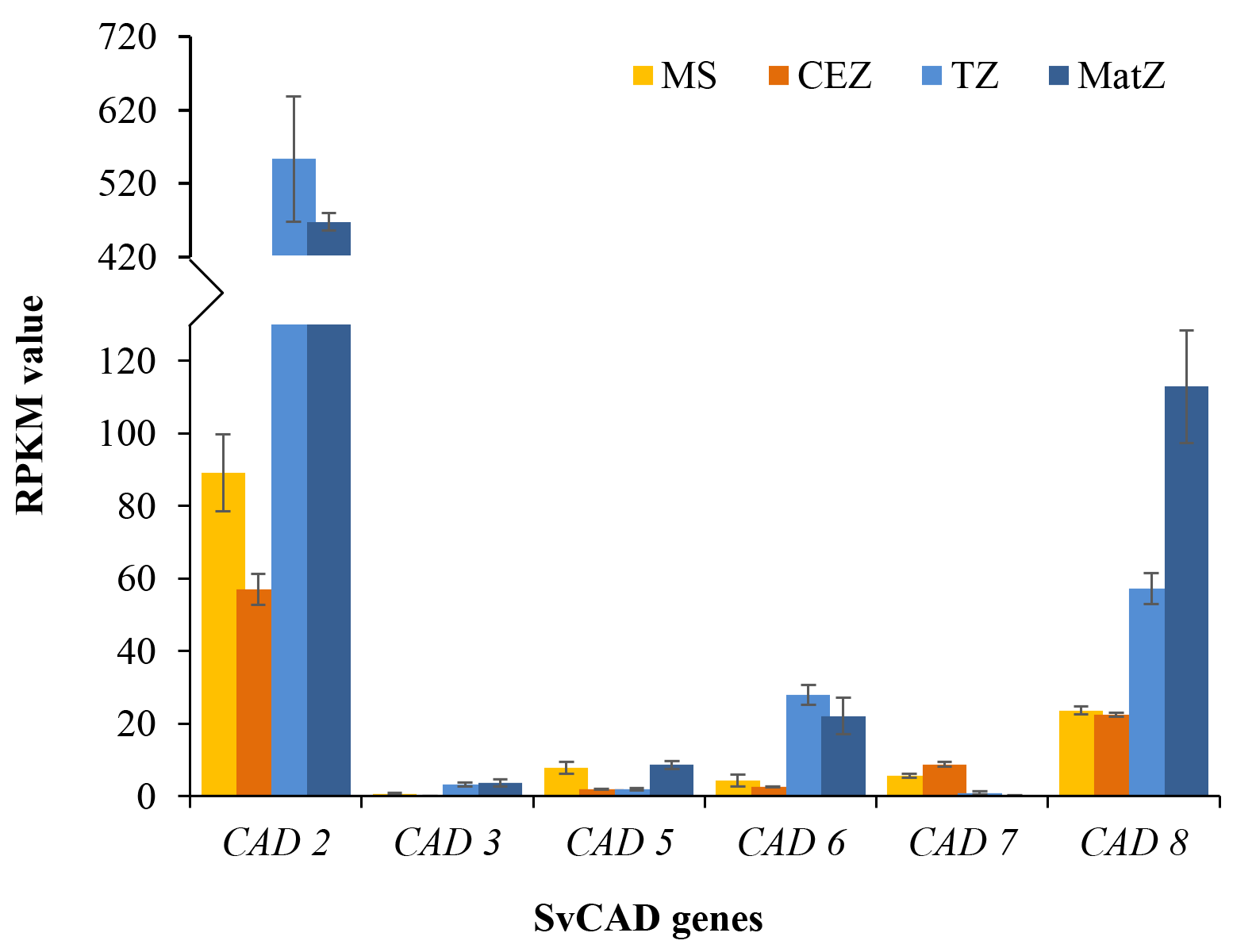


**Figure S2: Analysis of *Setaria viridis* *CAD* gene expression according to the RNA-Seq dataset published in [1].** The transcript abundance of each of the detected members of the *S. viridis CAD* gene family across the four developmentally distinct zones of the elongating internode, internode 5, according to the dataset published by Martin *et al.* [1]. Of the six *CAD*s identified from the [1] dataset, the *SvCAD2* (*Sevir.1G056800*) transcript was determined to be the most abundance in internode 5 and further, *SvCAD2* returned an expression profile expected of a gene that encodes a protein that plays a functional role in the formation of secondary cell walls, that is; less abundant in young or undifferentiated tissues and with a greatly enhanced abundance in transitioning and/or mature tissues. RPKM value: Reads Per Kilobase per Million mapped reads.

1. Martin AP, Palmer WM, Brown C, Abel C, Lunn JE, Furbank RT, et al. A developing *Setaria viridis* internode: an experimental system for the study of biomass generation in a C_4_ model species. Biotechnol Biofuels. 2016;9:45-57. doi: 10.1186/s13068-016-0457-6.
