# Supplementary material for: Reference gene identification for reliable normalisation of quantitative RT-PCR data in Setaria viridis
Source: Plant Methods. 2018 Mar 21;14:24. doi: 10.1186/s13007-018-0293-8 (PMC5861610; doi:10.1186/s13007-018-0293-8)
Supplement: Supplementary file 6 — Additional file 6: Figure S3. Profiling of the expression of SvCAD3, SvCAD5, SvCAD6 and SvCAD7 following normalisation with the most suitable, and the least suitable, set of reference genes. A–D RT-qPCR data to profile the expression of SvCADs, SvCAD3 (A; Sevir.6G025000), SvCAD5 (B; Sevir6G024400), SvCAD6 (C; Sevir7G014100), and SvCAD7 (D; Sevir7G245600) was normalised using the set of the three most suitable reference genes (ASPR6, DUSP and PP2A) and with the set of the three least suitable reference genes (FBoxD, PGM and SEIPIN). [file 13007_2018_293_MOESM6_ESM.docx]

Additional file 6

Reference gene identification for reliable normalisation of quantitative RT-PCR data in *Setaria viridis*

Duc Quan Nguyen^1^, Andrew L. Eamens^1†^ and Christopher P. L. Grof^1*†^

^1^ Centre for Plant Science, School of Environmental and Life Sciences, University of Newcastle, University Drive, Callaghan, NSW 2308, Australia

*** Correspondence:**Christopher Grof
[chris.grof@newcastle.edu.au](mailto:chris.grof@newcastle.edu.au)

^†^ These authors contributed equally to this work


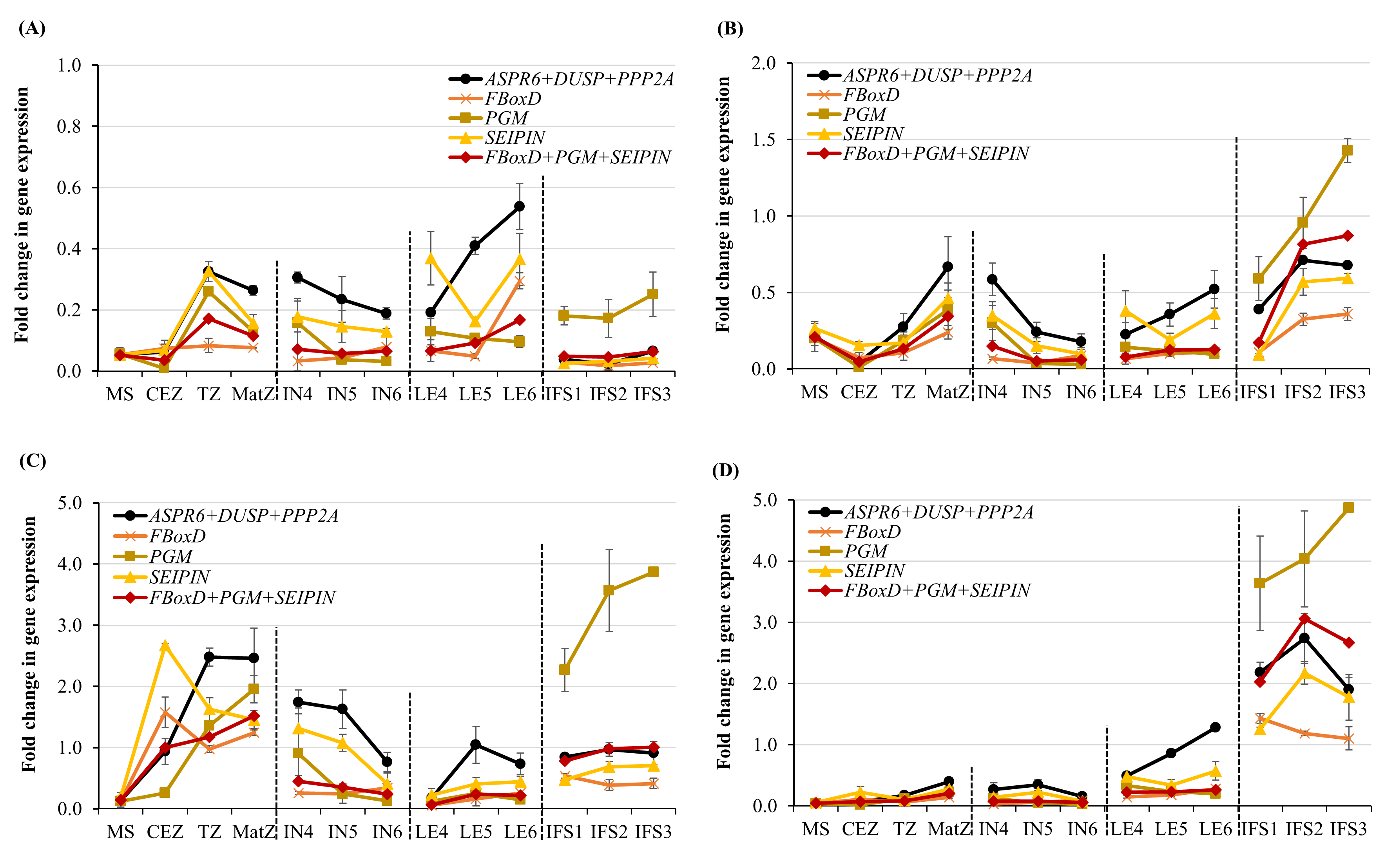


**Figure S3:** **Profiling of the expression of *SvCAD3, SvCAD5, SvCAD6 and SvCAD7* following normalisation with the most suitable, and the least suitable, set of reference genes. (A** to **D)** RT-qPCR data to profile the expression of *SvCAD*s, *SvCAD3* (**A**; *Sevir.6G025000*), *SvCAD5* (**B**; *Sevir6G024400*), *SvCAD6* (**C**; *Sevir7G014100*), and *SvCAD7* (**D**; *Sevir7G245600*) was normalised using the set of the three most suitable reference genes (*ASPR6*, *DUSP* and *PP2A*) and with the set of the three least suitable reference genes (*FBoxD*, *PGM* and *SEIPIN*). **MS**: Meristematic; **CEZ**: Elongating; **TZ**: Transitional; **MatZ**: Maturation zones; **IN4-6**: internode 4-6; **LE4-6**: leaf 4-6; **IFS1-3**: Inflorescence stage 1-3.
